# Iodine Promotes Glucose Uptake through Akt Phosphorylation and Glut-4 in Adipocytes, but Higher Doses Induce Cytotoxic Effects in Pancreatic Beta Cells

**DOI:** 10.3390/biology13010026

**Published:** 2024-01-01

**Authors:** Reséndiz-Jiménez Arely, Arbez-Evangelista Cristian, Arroyo-Xochihua Omar, Palma-Jacinto José Antonio, Santiago-Roque Isela, De León-Ramírez Yeimy Mar, Hernández-Domínguez Xcaret Alexa, Arroyo-Helguera Omar

**Affiliations:** 1 Centro de Investigaciones Biomédicas, Universidad Veracruzana, Av. Luis Castelazo Ayala S/N, Col. Industrial Ánimas, Xalapa 91190, Veracruz, Mexicoomar.arroyo98@hotmail.com (A.-X.O.); 2 Laboratory of Biochemistry and Neurotoxicology, Faculty of Bioanalysis-Xalapa, Universidad Veracruzana, Médicos y Odontólogos S/N, Unidad del Bosque, Xalapa 91190, Veracruz, Mexico; qbp_japj@hotmail.com (P.-J.J.A.); isantiago@uv.mx (S.-R.I.); 3 Laboratorio de Biomedicina y Salud Pública, Instituto de Salud Pública, Universidad Veracruzana, Av. Luís Castelazo Ayala S/N, Col. Industrial Animas, Xalapa 91190, Veracruz, Mexico; yeimy_mar@hotmail.com (D.L.-R.Y.M.);

**Keywords:** iodine, pancreatic damage, apoptosis, Glut4

## Abstract

**Simple Summary:**

Iodine is an essential trace element for thyroid hormone synthesis and plays a key role in extrathyroidal tissues. Recently, diabetes mellitus type 2 and elevated blood glucose have been associated with iodine excess. This study aims to elucidate the effect of iodine in mature adipocytes and pancreatic beta cells.

**Abstract:**

Background: Epidemiological clinical reports have shown an association between iodine excess with diabetes mellitus type 2 and higher blood glucose. However, the relationship between iodine, the pancreas, adipose tissue, and glucose transport is unclear. The goal of this study was to analyze the effect of iodine concentrations (in Lugol solution) on glucose transport, insulin secretion, and its cytotoxic effects in mature 3T3-L1 adipocytes and pancreatic beta-TC-6 cells. Methods: Fibroblast 3T3-L1, mature adipocytes, and pancreatic beta-TC-6 cells were treated with 1 to 1000 µM of Lugol (molecular iodine dissolved in potassium iodide) for 30 min to 24 h for an MTT proliferation assay. Then, glucose uptake was measured with the fluorescent analog 2-NBDG, insulin receptor, Akt protein, p-Akt (ser-473), PPAR-gamma, and Glut4 by immunoblot; furthermore, insulin, alpha-amylase, oxidative stress, and caspase-3 activation were measured by colorimetric methods and the expression of markers of the apoptotic pathway at the RNAm level by real-time PCR. Results: Low concentrations of Lugol significantly induce insulin secretion and glucose uptake in pancreatic beta-TC-6 cells, and in adipose cells, iodine-induced glucose uptake depends on the serine-473 phosphorylation of Akt (p-Akt) and Glut4. Higher doses of Lugol lead to cell growth inhibition, oxidative stress, and cellular apoptosis dependent on PPAR-gamma, Bax mRNA expression, and caspase-3 activation in pancreatic beta-TC-6 cells. Conclusions: Iodine could influence glucose metabolism in mature adipocytes and insulin secretion in pancreatic beta cells, but excessive levels may cause cytotoxic damage to pancreatic beta cells.

## 1. Introduction

The etiology of diabetes mellitus type 2 (DM2) involves insulin resistance in both muscle and liver, with glucose intolerance and high blood glucose levels (hyperglycemia) [1]. Metabolic abnormalities in carbohydrates, lipids, and protein metabolism are associated with insulin alterations in individuals with obesity [2]. Insulin resistance results in modifications to the insulin signaling pathway, decreased translocation of glucose transporters 4 (Glut4 in the cell membrane), and other molecular modifications [3]. Although the exact mechanism between insulin receptors and Glut4 is not known, the classic pathway PI3K/Akt is a key factor in regulating glucose metabolism [4]. The protein kinase Akt is regulated by tyrosine phosphorylation in threonine 308 and serine 473 residues and plays an important role in the expression and translocation of Glut4 to the cell membrane; therefore, Akt alteration is highly associated with the development of insulin resistance [4,5].

Marine algae consumption, such as sea tangle, sea mustard, and *Sargassum polycystum*, reduces plasma cholesterol and triglyceride levels and participates in controlling glycemic levels [6,7]. Brown seaweeds and kelps accumulate high concentrations of iodine and are part of the Asian diet [8]. The iodine effect involves antioxidant, apoptotic, and anti-proliferative mechanisms activated by the formation of iodolipids (IL) from unsaturated fatty acids such as arachidonic acid (AA), which are ligands for nuclear receptors named PPAR-γ [9,10]. Recently, we demonstrated for the first time in mature 3T3-L1 adipocytes the presence at the mRNA level of pendrine (PEN) and the symporter sodium-iodine (NIS), as well as the lipolytic effect induced by iodine through the regulation of PPAR-γ [11]. PPAR-γ is known as a “master gene” that regulates a wide range of processes in adipose tissue, including the signal transduction of insulin, lipid and glucose metabolism, the secretion of adipokines, apoptosis, and adipocyte differentiation [12,13]. In this regard, it has been reported in mammary and thyroid glands that iodine supplementation regulates the expression of PPAR-γ through the formation of iodinated lipids to maintain the normal physiology of the mammary gland [14,15].

DM-2 has a multifactorial etiology, and recently, high iodine levels (>221 µg/L) have been associated with DM-2 [15,16]. High glucose levels and blood pressure have been associated with DM-2 without alterations in their thyroid profile [16]. In addition, a urinary iodine concentration (UIC) between 300 and 799 μg/L was associated with metabolic syndrome and impaired glucose tolerance, and a urinary iodine concentration between 500 and 799 μg/L was related to prediabetes [17]. Contrasting data have also been found. For example, iodine and 25(OH)D levels were much lower in T2DM patients than in control subjects [18], and iodine deficiency is associated with an increase in the risk of diabetic kidney disease, independent of thyroid function in diabetic patients [19]. However, it is known that high levels of iodine can induce oxidative damage and apoptosis [10,20,21], which suggests that pancreatic and adipose tissue could be targets of iodine, and the effects of iodine on the insulin-mediated glucose transport pathway have not been described. The goal of this study was to analyze the effect of several concentrations of iodine (as Lugol) on cytotoxicity mechanism, glucose transport, and insulin secretion in pancreatic beta-TC-6 cells, as well as its effects on glucose uptake mediated by the insulin-activated IR/Akt/p-Akt/Glut4 signaling pathway in mature 3T3-L1 adipocytes.

## 2. Materials and Methods

### 2.1. Culture Cells and Adipocytes Cell Differentiation

3T3-L1 fibroblast and pancreatic beta-TC-6 cell lines were initially grown in Dulbecco’s Modified Eagle Medium (DMEM) supplemented with 8% fetal bovine serum in 5% CO_2_ atmosphere at 37 °C. After reaching a confluence of 70%, 3T3-L1 fibroblasts were differentiated to adipose cells using a differentiation medium containing 1 µM of dexamethasone (PiSA), 0.5 mM isobutyl-1-methylxanthine (Sigma, St. Louis, MO, USA) and 10 µg/mL insulin (Sigma). After 72 h in differentiation medium, the cells were incubated between 4 and 5 days in medium DMEM supplemented with 8% fetal bovine serum and 10 µg/mL of insulin. On the seventh day, more than 90% of cells showed morphology of mature adipocytes.

The experiments were done in pancreatic beta-TC-6 cells and mature adipocytes treated with 0, 1, 10, 100, 500, and 1000 µM of Lugol (molecular iodine dissolved in potassium iodide) for 0.5, 6, and 24 h. 3T3-L1 fibroblasts were used as the control cell line because they do not express NIS mRNA transporter.

### 2.2. Viability MTT Assay, LD50 Calculation, Alpha-Amylase, and Caspase-3 Activities

Mature adipocytes and pancreatic beta-TC-6 cells were seeded in 6-well plates (30 × 10^3^ cells/well). After 24 h, the medium was changed, and the cells were incubated with different concentrations of Lugol during 0, 6, and 24 h. Then, after treatment, the cells were incubated in the dark with an MTT solution for 4 h at 37 °C. The supernatant was eliminated, and 100 μL of DMSO was added to dissolve the formazan crystal product. Absorbance was then measured at 570 nm, and fibroblast 3T3-L1 cells were used as control just in MTT assay (this cell line does not capture iodine since it does not express iodine transporters). The alpha-amylase activity was measured by colorimetric assay and caspase-3 activity following the manufacturer’s instructions using the Caspase-3 Colorimetric Assay Kit (ab39401). This is based on the formation of the chromophore p-nitroaniline (pNA) by cleavage from the labeled substrate DEVD-pNA. The pNA was quantified using a microtiter plate reader at 405 nm. The absorbance was measured using an Epoch microplate (Biotek, Winooski, VT, USA). Median inhibitory concentrations (IC50) were statistically analyzed using GraphPad Prism Version 6 software (GraphPad Software, 2016). Non-linear regressions of log (inhibitor) vs. response with four parameters were selected for IC50 estimation.

### 2.3. Oxidative Stress and Acridine Orange/Ethidium Bromide Fluorescent Staining

Oxidative stress was measured by the concentration of lipid peroxidation product malondialdehyde (MDA) or thiobarbituric acid reactive substances (TBARS) by colorimetric assay. TBARS were measured in 90 μL of protein sample, which was mixed with TRIS (150 mM pH 7.5), 0.4% of tiobarbituric acid, 20% acetic acid pH 3.0, and then, all samples were warmed to 100 °C during 45 min. The samples were cooled in ice, and 1.2% of KCl was added. After centrifugation, overnadants were read at 532 nm in a microplate reader (Spectramax Plus; Molecular Devices, Sunnyvale, CA, USA). The data were expressed in nmol MDA/mg protein. Acridine orange/ethidium bromide (AO/EB) staining was used to visualize nuclear changes and apoptotic body formation that are characteristic of apoptosis. Briefly, the cells were seeded at 2 × 10^4^/well. After treatments and 24 h of incubation, the supernatant (medium and floating cells) was transferred to 1.5 mL tubes. The rest of the adherent cells were detached with PBS-EDTA 1 mM (Invitrogen, Grand Island, NY, USA). The supernatant and the detached cells were pooled and pelleted by centrifuging at 1000 RPM for 5 min and washed twice with 1 mL of cold PBS. Cell pellets were re-suspended in 25 μL cold PBS and stained with 1 μL EB/AO dye mix containing 100 μg/mL AO and 100 μg/mL EB (AO/EB, Sigma, St. Louis, MO, USA). Stained cell suspensions (10 μL) were placed on a clean microscope slide and covered with a coverslip. Cells were viewed, and photographs were taken on a fluorescent inverted microscope (Motic, Barcelona, Spain, AE31) with a 480/30 nm excitation filter, dichromatic mirror cut-on 505 nm LP, and 535/40 nm barrier filter. Pictures were taken with a Motic PRO camera and Imagine Image Plus 2.0 mL 2017 software. Tests were conducted in triplicate.

### 2.4. Measurement of Glucose Uptake and Insulin Levels

Measurement of glucose uptake was performed using 2-[*N*-(7-nitrobenz-2-oxa-1,3-diazol-4-yl)] amino-2-deoxy-D-glucose (2-NBDG) fluorescent analog (Sigma–Aldrich, St. Louis, MO, USA). In brief, adipocytes were starved and depleted of glucose in a phenol-red-free minimum essential medium (MEM) without serum bovine serum (FBS) overnight in an atmosphere of 5% CO_2_ at 37 °C. Then, the cells were washed with cold PBS, and different concentrations of Lugol (1–1000 µM) and 0.05 mL of 2-NBDG analog were added to a final concentration of 50 µM. In addition to this test, the combined effect of Lugol and insulin 50 nM was evaluated. After 1 h at 37 °C, the reaction was stopped by washing with cold PBS, and quickly, the cells were lysed with 0.1 mL of Buffer Salt (40 mM KCl, 20 mM Tris, 1% Triton X-100 and Protein Inhibitor Cocktail) (Sigma–Aldrich). The content of each well was centrifuged at 3500 rpm/−4 °C for 5 min, the supernatant was placed in a 96-well plate, and the fluorescence of 2-NBDG was measured using a wavelength of 551 nm (excitation wavelength at 494 nm). The treated cells were pre-incubated with Krebs–Ringer bicarbonate HEPES buffer (KRBH: 115 mM NaCl, 5 mM KCl, 1 mM MgCl_2_, 24 mM NaHCO_3_, 2.5 mM CaCl_2_, 10 mM HEPES) supplemented with 1% FBS and a low level of glucose (3.0 mM) for 1 h. Afterward, the cells were incubated in KRBH buffer containing 3.3 or 16.7 mmol/L glucose for 1 h (this dose was used to measure a moderate (5.6 mM) or high (16.7 mM) glucose-induced insulin secretion), then Lugol treatment was performed for 2 h, and the supernatants were collected, and insulin concentrations were measured using an insulin enzyme-linked immunosorbent assay. The insulin level secretion was normalized to the protein-level measurement using the Bradford method.

### 2.5. Western Blotting

The adipose cells were treated with 1, 5, 10, and 100 µM of Lugol for 30 min and 6 h (higher Lugol doses were discarded because they inhibited cell proliferation), then cells were washed with cold PBS and lysed with 0.1 mL RIPA Buffer (1% Triton X-100, 150 mM NaCl, 0.1% SDS, 50 mM Tris-HCl pH 7.6, 10 mM EDTA, 1 mM PMSF, and 10 µg/mL of Cocktail Protease Inhibitor). The protein concentration was measured using the Bradford method. Lysates (50 µg protein) under reducing conditions were directly subjected to SDS-PAGE (10% gels) followed by Western blotting with antibodies against insulin receptor (ab5500), Glut4 (ab654) from Abcam, Cambridge, MA, USA, Akt (mAb#4691), and p-Akt ser-473 (antibody #9271) from Cell Signaling Biotechnologies at dilutions of 1:1000 in TBST containing 10% BSA and β-actin (Cell Signaling Biotechnologies). The PVDF membranes were incubated overnight at 4 °C with primary antibodies. Thereafter, three washes were performed with TBST, and membranes were incubated with a secondary antibody (Cell Signaling, Danvers, MA, USA, 65-6120) diluted in TBST with BSA at 10% (1:4000) for 1h at room temperature. The signal was detected using a 1:1 solution from Amersham ECL Prime Western Blotting (Thermo Fisher Scientific Inc., Munich, Germany) and visualized on the Gel-Doc (BioRad, Hercules, CA, USA). The relative OD ratio was calculated with the ImageJ 1.52a program. Data were normalized with β-actin to the insulin receptor, Glut4, and total Akt, and p-Akt with total Akt protein; data from three experiments were analyzed.

### 2.6. Determination of mRNA by RT-PCR

Total RNA extraction was performed with TriReagent (Sigma–Aldrich). Cells in Petri dishes were spiked with TriReagent and incubated at room temperature. The sample was transferred to 1.5 mL Eppendorf tubes, and chloroform was added. It was manually mixed and incubated for 10 min at room temperature, followed by centrifugation at 12,000 rpm for 5 min at 4 °C. The aqueous phase was transferred to another Eppendorf tube, and isopropanol was added, mixed again, and incubated for 10 min, then centrifuged at 12,000 rpm for 10 min at 4 °C. The pellet obtained was washed 3 times with 75% cold ethanol. Between each wash, it was centrifuged at 7500 rpm for 5 min at 4 °C. The ethanol was allowed to evaporate, and the pellet was re-suspended in sterile water. RNA quantification was performed on a multiple-volume spectrophotometer, using only 2 µL of the sample at an optical density reading at 260 and 280 nm to measure nucleic acids and proteins, respectively. RNA integrity was analyzed by horizontal electrophoresis in 2% agarose gel with ethidium bromide as a fluorescence marker. The mRNA was synthesized to cDNA using ReverTra Ace^®^ qPCR RT master mix. Quantitative real-time PCR was conducted with a SensiFAST™ SYBER^®^ No-ROX Kit (SIGMA) in a Piko Real-Time PCR System (Thermo Sci, Boston, MA, USA). PPAR-γ forward: 5′-TGCCAGTTTCGATCCGTAGA-3′, reverse 5′-AGGAGCTGTCATTAGGGACATC-3′; P53 forward 5′-GTATTTCACCCTCAAGATCC-3′, reverse 5′-TGGGCATCCTTTAACTCTA-3′; Bax forward 5′-CTACAGGGTTTCATCCAG-3′, reverse 5′-CCAGTTCATCTCCAATTCG-3′; Bcl-2 forward 5′-GTGGATGACTGAGTACCT-3′, Bcl-2 reverse 5′-CCAGGAGAAATCAAACAGAG-3′; GAPDH forward 5′-GGCCATCCACAGTCTTCTGG-3′, reverse 5′-ACCACAGTCCATGCCATCACTGCCA-3′. Denaturation was carried out at 94 °C for 3 min, and the next 40 cycles were 35 s at 94 °C, 30 s at 58–60 °C and 30 s at 72 °C. The relative expression was calculated to the housekeeping gene GAPDH normalized as an internal control with the 2^−ΔΔCT^ method.

### 2.7. Statistical Analysis

The data in the figures are represented as the mean ± standard deviation (SD) of independent experiments (n = 6 and n = 3). Differences between groups were obtained using a one-way ANOVA, followed by the Dunnett test using GraphPad Prism software (GraphPad Software Inc., San Diego, CA, USA). Values of *p* < 0.05 were considered statistically significant.

## 3. Results

### 3.1. High Iodine Levels Induce Cytotoxic Effects by Increasing Oxidative Stress and Apoptosis in Mature Adipocytes and Pancreatic Beta-TC-6 Cells

**Figure 1:**
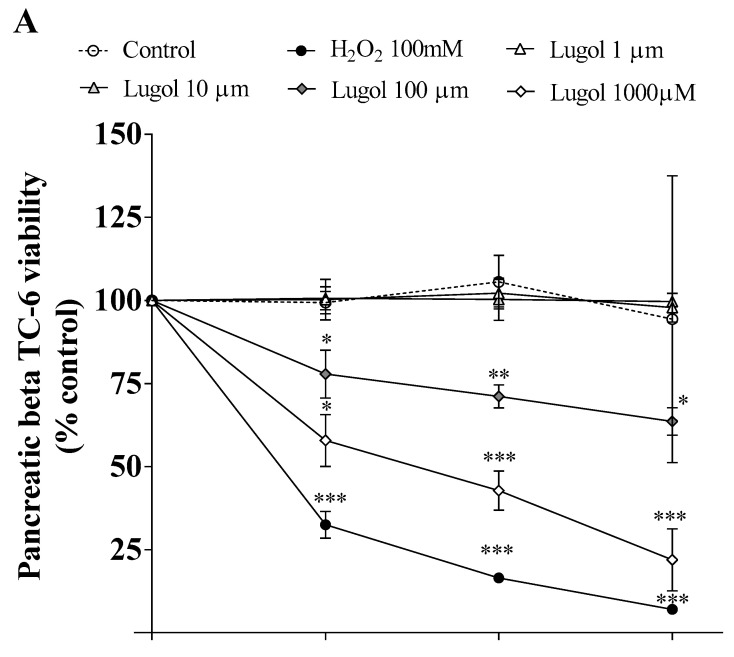

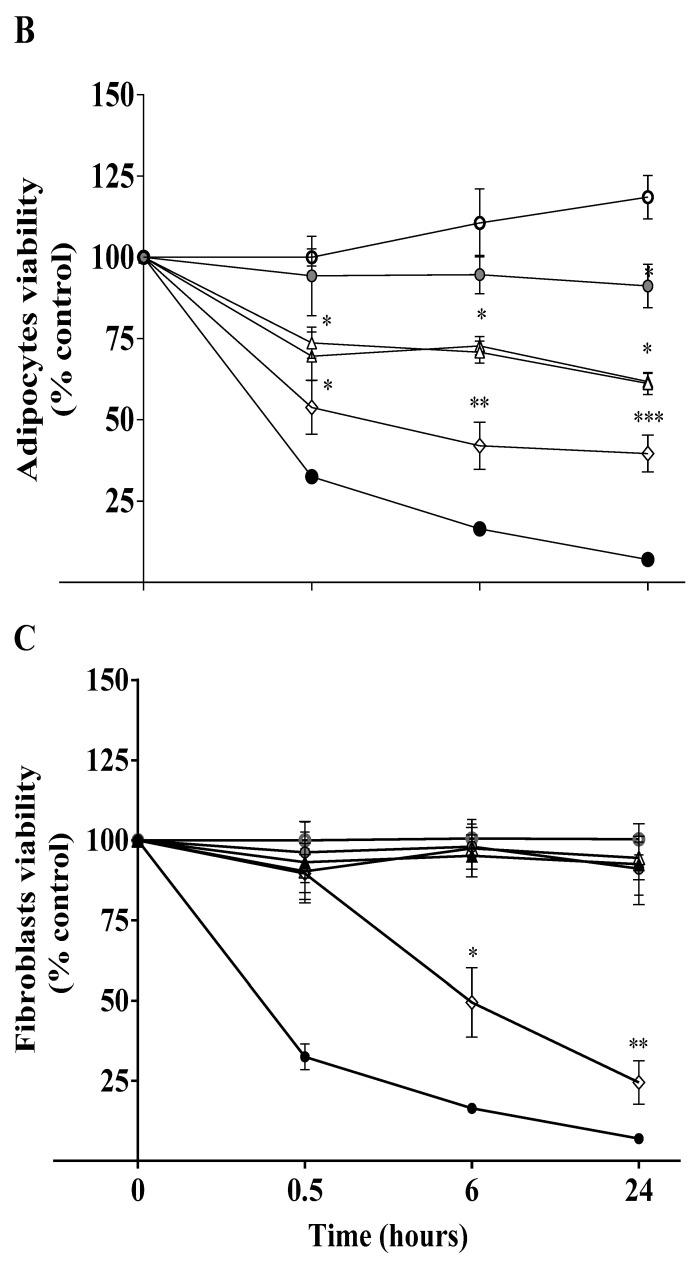
Cell viability in pancreatic beta-TC-6 cells, adipocytes, and fibroblast cells. MTT assay was conducted to measure cell viability. (**A**) the viability of pancreatic beta-TC-6 cells; (**B**) viability of mature adipocytes; and (**C**) fibroblast cells (do not uptake iodine). Data were normalized to control cells. * *p* < 0.05; ** *p* < 0.01; *** *p* < 0.01 compared with control cells (vehicle). Each assay represents an independent experiment performed in triplicate, n = 5. Data are presented as mean ± SD from three experiments with duplicate determination. * *p* < 0.05, ** *p* < 0.01, *** *p* < 0.001 vs. control group.

Adipocytes and pancreatic beta-TC-6 cells were treated with 1–1000 µM of Lugol for 0.5, 6, and 24 h. As presented in Figure 1A,B, it is observed that Lugol inhibited the proliferation in a dose and time-dependent effect since 10 and 100 µM of Lugol in adipocytes and pancreatic beta-TC-6 cells, compared to the control group (*p* < 0.05); by contrast, fibroblast control cells (cells that do not express iodine transporters) at doses of 1–100 µM cell proliferation decrease was not observed (Figure 1C). However, doses higher than 1000 µM Lugol decrease the proliferation of all cells (Figure 1A–C).

**Figure 2:**
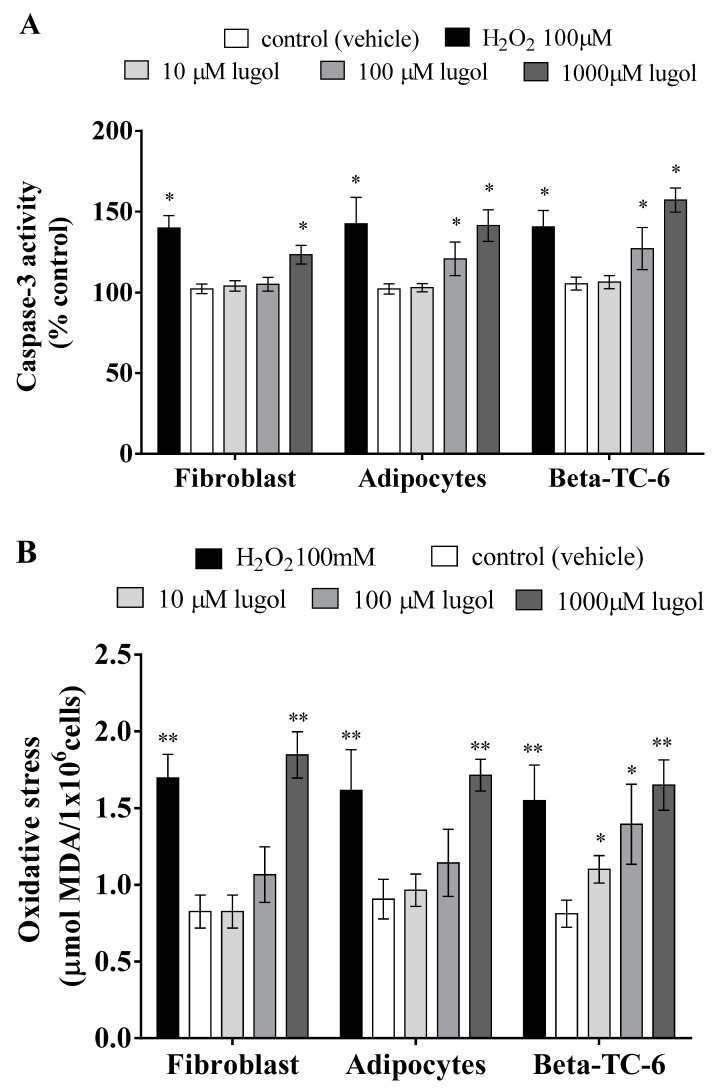
Lugol induces caspase-3 activity and oxidative stress in fibroblast, mature adipocytes, and pancreatic beta-TC-6 cells after about 24 h of exposition. The cells were exposed to 10, 100, and 1000 µM of Lugol at 24 h. (**A**) caspase-3 activity; (**B**) oxidative stress. Data were normalized in control cells. * *p* < 0.05; ** *p* < 0.01 compared with control cells (vehicle). Each assay represents an independent experiment performed in triplicate, n = 4. Data are presented as mean ± SD from three experiments with duplicate determination. * *p* < 0.05, ** *p* < 0.01, vs. control group.

In Figure 2, the results show that at 24 h of treatment with 100 and 1000 µM of Lugol, there is increasing caspase-3 activity and high oxidative stress (TBAR levels) in adipocytes, pancreatic beta-TC-6 cells, and fibroblast cells (Figure 2A,B). However, it is observed that pancreatic beta-TC-6 cells and mature adipocytes were more sensitive to higher Lugol concentrations (Figure 2A,B).

**Figure 3:**
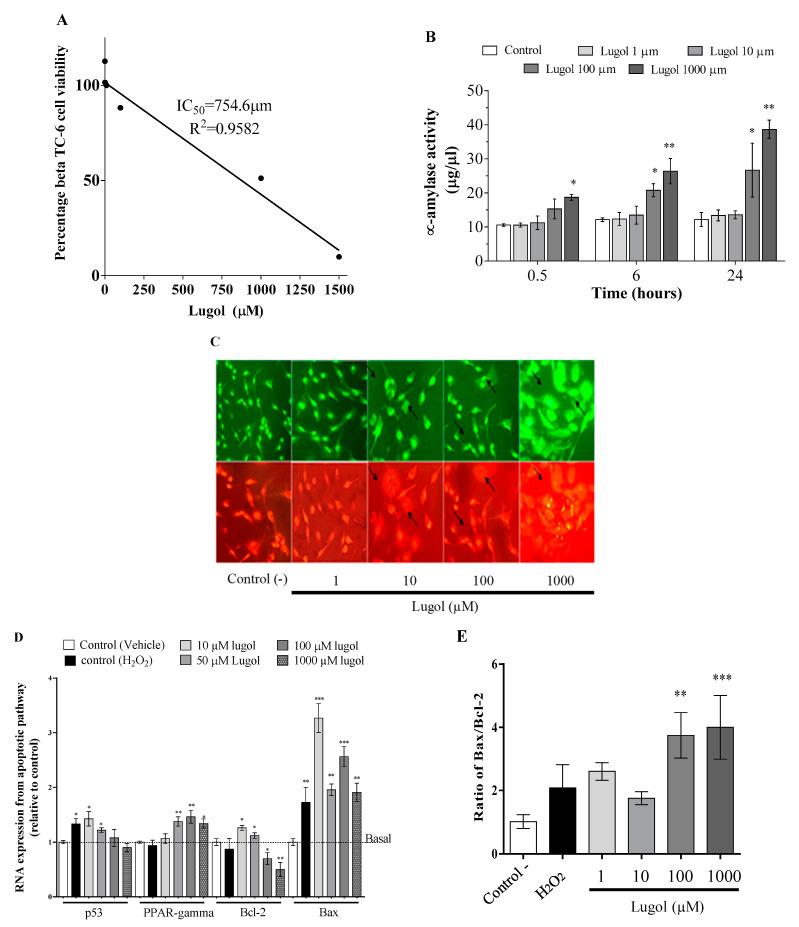
Cytotoxic mechanism mediated by Lugol in pancreatic beta-TC-6 cells. The cells were treated with different concentrations of Lugol (1–100 µM of Lugol) in a serum-free medium. (**A**) LD50 was calculated with linear regression with data from MTT assay at 24 h of Lugol exposition with doses from 1 to 1500 µM; (**B**) α-amylase activity was measured as a pancreatic damage marker; (**C**) representative photograph from pancreatic cells stained with EB(red)/AO(green) to visualize apoptotic characteristic—arrows indicate cells with pyknotic and karyorrhexis apoptotic characteristics; (**D**) after about 6 h of Lugol treatment, total ARN was extracted and relative mRNA expression of p53, PPAR-γ, Bcl-2, and Bax were detected by qRT-PCR; (**E**) the effect of Lugol on the Bax/Bcl2 index. Data are mean ± SD values from six experiments by triplicate determination. * *p* < 0.05, ** *p* < 0.01, *** *p* < 0.001 vs. control adipocytes (vehicle) (n = 6). The scale bar is 100 µm.

Figure 3 shows the cytotoxic effect of Lugol in pancreatic beta-TC-6 cells. The IC50 calculated was 754 μm of Lugol (Figure 3A). Figure 3B shows that Lugol increases the activity of α-amylase (a pancreatic damage marker), as it is significant at 6 h with 100 and 1000 µM in comparison with control cells and with high doses of 1000 µM at 0.5 h of treatment (Figure 3B). In Figure 3C, cellular pyknotic and karyorrhexis are observed with 100 µM Lugol. These are apoptotic characteristics after about 6 h of treatment. Previous reports have suggested that iodine can induce apoptosis via PPAR-γ. Figure 3D illustrates an increase in the mRNA expression of PPAR-γ, Bax, and Bax/Bcl-2 index (Figure 3E) after about 24 h with 100 and 1000 µM of Lugol in pancreatic beta-TC-6 cells.

### 3.2. Effect of Low Doses of Lugol on Insulin Secretion in Pancreatic Beta-TC-6 Cells and Glucose Transport in Mature 3T3-L1 Adipocytes

**Figure 4:**
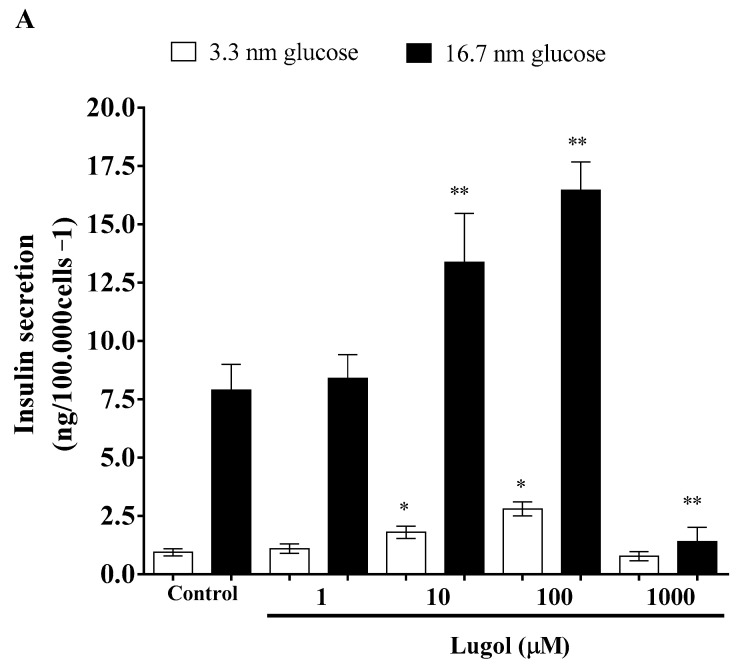

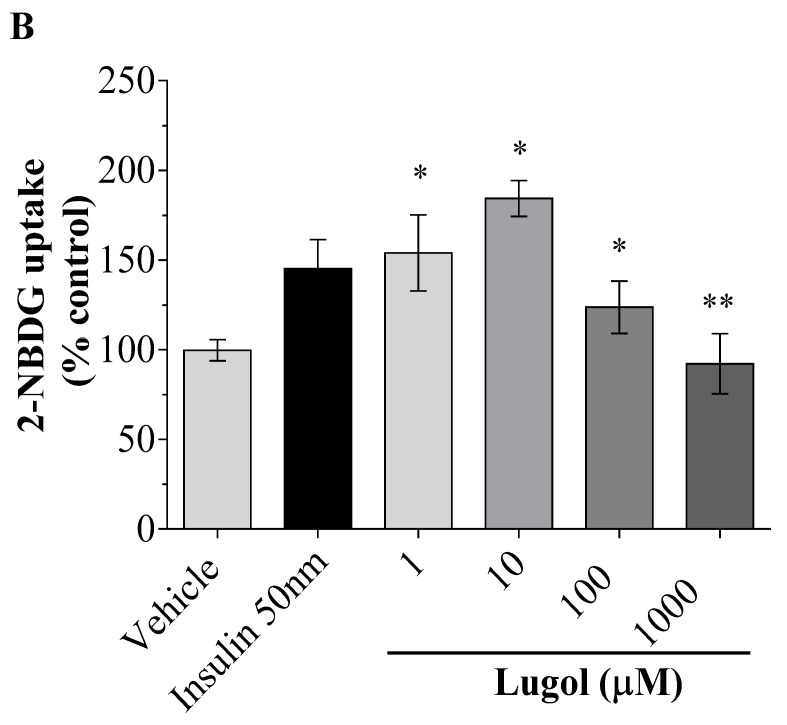
Effect of Lugol treatment on insulin secretion in pancreatic beta-TC-6 cells under low (3.3 mmol/L) and high (16.7 mmol/L) glucose conditions and glucose uptake in mature adipocytes. (**A**) pancreatic beta-TC-6 cells and adipocytes were exposed to 1 to 1000 µm of Lugol for 2 h, and insulin levels were determined by colorimetric methods; (**B**) mature adipocytes were treated with serum-free medium with 1–1000 µM of Lugol for 30 min and glucose uptake by 2-NBDG assay. Data are presented as mean ± SD from three experiments with duplicate determination. * *p* < 0.05, ** *p* < 0.01 vs. control group (n = 3).

To know the effect of Lugol on insulin secretion, we used 1 to 1000 µM of Lugol in pancreatic beta-TC-6 cells stimulated with low (3.3 mmol/L) and high glucose (16.7 mm/L). The results show that 1, 10, and 100 µM of Lugol increased insulin secretion in comparison with untreated cells (*p* < 0.05). Despite higher doses (1000 µM of Lugol) not inducing insulin secretion (Figure 4A), this could be by proliferation inhibition (as seen in previous figures) or insulin stimulation decrease. Related to glucose transport, we use the mature adipocytes, and, as shown in Figure 4B, low doses of Lugol (1 and 10 µM) increased glucose transport by 41.71, and 36.05%, respectively (*p* < 0.001 vs. control adipocytes) after 30 min of treatment, and, with 100 µM of Lugol, the uptake of glucose was similar that of control cells (vehicle), even decreasing significantly with 100 and 1000 µM of Lugol in comparison with control cells (Figure 4B).

### 3.3. The Mechanism Involved in the Glucose Uptake Mediated by Lugol in Mature Adipocytes

**Figure 5:**
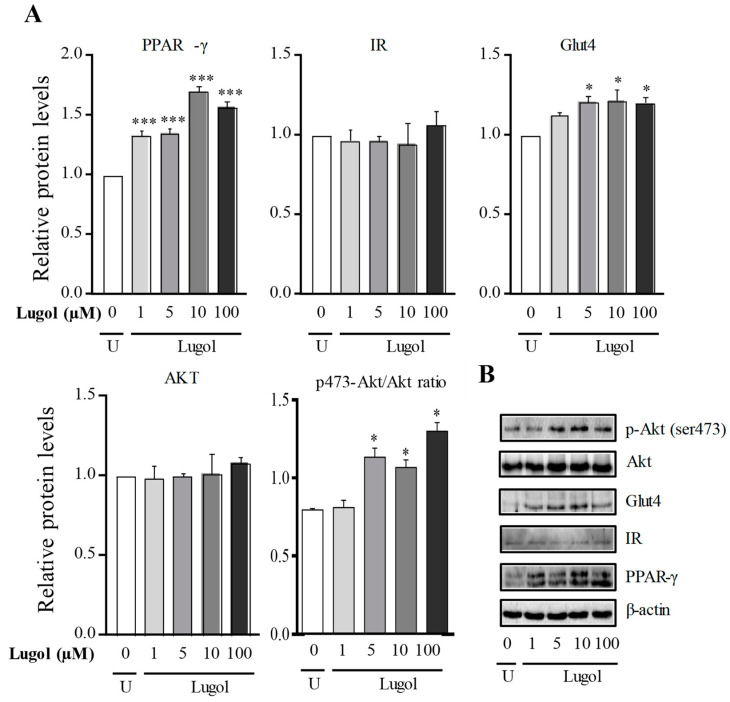
Effect of Lugol on the regulation of glucose-uptake pathway in mature adipocytes after 30 min of treatment. (**A**) densitometric analysis from untreated cells (U: untreated control cells) and treated with Lugol iodine after about 30 min; (**B**) representative Western blot protein bands. Mature 3T3-L1 adipocytes were treated with Lugol (1–100 µm) for about 30 min and lysed with RIPA buffer. The protein extract was subject to SDS-PAGE 10%, and the blots were incubated with the primary and secondary antibodies. Detection was performed with the chemiluminescence protocol. Data were normalized with β-actin levels and its reported relative protein levels to the insulin receptor, Glut4, and total Akt, or p-Akt with total Akt protein (ratio levels). Data represented mean ± SD, n = 3. * *p* < 0.05, *** *p* < 0.001 vs. control cells. The uncropped western blot figures were presented in Figures S1–S4.

**Figure 6:**
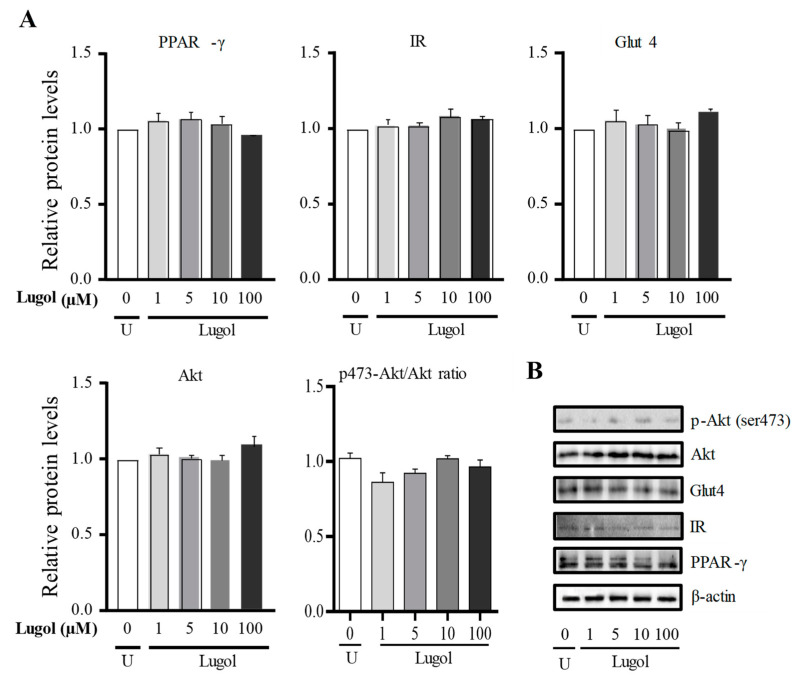
Effect of Lugol on the regulation of glucose-uptake pathway in mature adipocytes after about 6 h of treatment. (**A**) densitometric analysis of untreated cells (U: untreated control cells) and treated with Lugol after about 6 h; (**B**) representative Western blot protein bands. Mature adipocytes were treated with Lugol at 1, 5, 10, and 100 µm (high doses of iodine were not used because they inhibit cell proliferation) for about 6 h and lysed with RIPA buffer. The protein extract was subject to SDS-PAGE 10%, and the blots were incubated with primary and secondary antibodies, as described in the methodology section. Detection was performed with the chemiluminescence protocol. Data were normalized with β-actin levels and its reported relative protein levels to the insulin receptor, Glut4, and total Akt, or p-Akt with total Akt protein (ratio levels). Data represented mean ± SD, n = 3. The uncropped western blot figures were presented in Figure S5–S8.

In Figure 5, we use doses of 1 at 100 µM to know the glucose-uptake mechanism (high Lugol doses were discarded because they inhibited cell proliferation). The results show that after about 30 min, a significant increase of PPAR-γ (*p* < 0.001) is observed, while at 6 h, no significant changes in PPAR-γ expression were observed (Figure 5). The insulin receptor (IR) and Akt protein expression do not change in comparison with control adipocytes after 30 min and 6 h of treatment. However, Glut4 expression increased significantly in groups treated with concentrations between 5 and 100 µM of Lugol for 30 min (*p* < 0.05 vs. control adipocytes) but not for 6 h (Figure 5 and Figure 6), the uncropped western blot figures were presented in Supplementary Materials.

Then, the Akt phosphorylated form was evaluated, which is required for full activation and dose, since 1 to 100 µM of Lugol exposition increased the Ser-473 Akt phosphorylated/Akt ratio in comparison with control adipocytes (*p* < 0.05) (Figure 5 and Figure 6), the uncropped western blot figures were presented in Supplementary Materials.

## 4. Discussion

In recent years, the number of individuals with insulin resistance and DM-2 has increased. Recently, high iodine levels (>221 µg/L excess) have been found in patients with DM-2 [15]. Also, high glucose levels and blood pressure have become associated with DM-2 without alterations in their thyroid profile, as reported by Liu et al., 2019 [16]. In addition, the relationship between urinary iodine concentrations (UIC) and metabolic diseases, for example, ranging from 300 to 799 UIC, is associated with metabolic syndrome, impaired glucose tolerance occurrence, and prediabetes occurrence [17]. The evidence suggests that iodine is involved in the regulation of glucose. Our first evaluation showed that iodine in Lugol solution affects glucose transport in mature adipocytes and insulin secretion in pancreatic cells. Our results showed that low doses of iodine in Lugol solutions increase the uptake of the glucose analog 2-NBDG uptake in adipocytes and induce the secretion of insulin in pancreatic beta-TC-cells. These results agree with the increase in glucose transport in adipose tissue of individuals who have been administered thiazolidinedione (TZDs), a group of drugs that act as PPAR-γ ligands [21,22] and have a similar mechanism of action to the Lugol [23]. In our study, we found that higher doses of Lugol induce proliferation inhibition, oxidative stress, and activation of the apoptotic pathway dependent on PPAR-γ and Bax expression in pancreatic beta-TC-6 cells. These data show that higher doses of iodine could be involved in pancreatic failure because higher doses increase alpha-amylase activity, considered a pancreatic damage marker. All these data and epidemiological data [15,16] indicate that higher doses of iodine could be involved in pancreatic damage. In addition, in MCF-7 cell lines, the formation of iodolactones acts as a PPAR-γ ligand that binds to specific DNA sequences [7,8,9] and then can activate the expression of genes associated with lipid and glucose metabolism or apoptosis [11]. This result supports our recent findings that Lugol induces a lipolytic effect dependent on PPAR-γ expression in mature adipocytes [12].

The glucose internalization in adipose tissue requires the mobilization of vesicles containing Glut4 from the cytoplasm to the cell membrane [24]. Although we did not evaluate the mobilization of Glut4 in this study, our results suggest that glucose transport is dependent on Glut4 expression. This could be related to PPAR-γ activation (which is abundant in adipose tissue) because this transcription factor has been shown to directly regulate the expression of Glut4 and c-Cbl proteins [25,26]. This is supported by our previous study in mature adipocytes 3T3-L1, which showed that treatment with iodine–Lugol solutions increased PPAR-γ expression [10]. Furthermore, the Glut4 promoter region −66/+163 pb contains response elements to PPAR (PPRE) that can modulate its expression [27]. On the other hand, exposure to Lugol for 30 min and 6 h did not significantly affect the total protein expression of the insulin receptor in adipocytes. These results lead us to hypothesize that there exists an increase in the activation of a β subunit of receptors because some reports have shown that hypoglycemic drugs increase the phosphorylation of insulin receptors and not its total expression [28,29]. In addition, there are no responsive elements for PPAR-γ in the promoter regions of insulin receptors [30]. Finally, our data reveal that iodine rapidly increases glucose uptake in adipocyte cells, which, at least for 30 min, depend on an increment in the Glut4 transporter to potentiate insulin action. We propose that this rapid iodine effect involves the translocation of the Glut4 transporter at the cell surface level. This is supported by the phosphorylation of Akt. Also, another Glut transporter may be involved, although these assumptions must be corroborated with more studies.

TZD has shown effects on insulin sensitivity at the Akt level in several models of obesity and diabetes, demonstrating the importance of this kinase in glucose metabolism [21]. According to our results, exposure to Lugol for 30 min and 6 h did not produce changes in the total expression of the Akt protein, although we show the activation of serine residues-473 from Akt (*p* < 0.05 vs. control). In this regard, some authors have demonstrated an increase in the activation of the phosphorylated form of Akt in diabetic patients treated with rosiglitazone [26,30]. Moreover, different investigations have demonstrated that the effects of TZDs in improving the activity of Akt and PI3K protein kinases are due to direct or indirect activation of PPAR-γ and its effect on gene expression [31]. For example, in muscle cells of individuals with T2D and obesity, troglitazone increases the expression of PPAR-γ at mRNA and protein levels, as well as increases the activity of glycogen synthase [32]. Because serine-473 phosphorylation of Akt can be considered to be an indirect downstream marker of PI3K protein activity [19], this suggests that iodine–Lugol solution will also improve the activity of this protein, an assumption that is supported by the fact that TZD can activate the p110 catalytic subunit of PI3K [31]. Another explanation may involve the phosphorylation of the AS160 protein on multiple sites by Akt, then AS160 phosphorylation is required for the translocation of GLUT4 to the plasma membrane [33]. In addition, the gene TBC1D4 that encoded the AS160 protein, a Rab-GTPase-activating protein, is regulated by ER-alpha, and iodine treatment can activate these receptors [34]. This suggests that AS160 could be participating in the translocation of Glut4 to the membrane, although this hypothesis should be studied.

## 5. Conclusions

Iodine at low doses stimulated glucose uptake through Akt phosphorylation at S473 and Glut4 expression in mature adipocytes and induced insulin secretion in pancreatic beta-TC-6 cells. However, higher iodine concentrations induced the loss of cell viability and increased the activity of alpha-amylase (pancreatic damage marker), high oxidative stress, and activation of the apoptosis pathway dependent on PPAR-γ in pancreatic beta-TC-6 cells. More research is necessary to corroborate the results and establish a detailed mechanism of action in animal models. Also, the cytotoxic effect of iodine excess in the function of the pancreas must be determined.

## Data Availability

All data included in this study are available in the main text and Supplementary Materials.

## Supplementary Materials

The supporting information can be downloaded at https://www.mdpi.com/article/10.3390/biology13010026/s1, Figures S1–S8: details of Western blot membranes.
