# Supplementary material for: Iodine Promotes Glucose Uptake through Akt Phosphorylation and Glut-4 in Adipocytes, but Higher Doses Induce Cytotoxic Effects in Pancreatic Beta Cells
Source: Biology (Basel). 2024 Jan 1;13(1):26. doi: 10.3390/biology13010026 (PMC10813031; doi:10.3390/biology13010026)
Supplement: Supplementary file 1 [file biology-13-00026-s001.zip › biology-2708511-supplementary.pdf]

Supplementary material

Figures S1–S8: details of western blot membranes

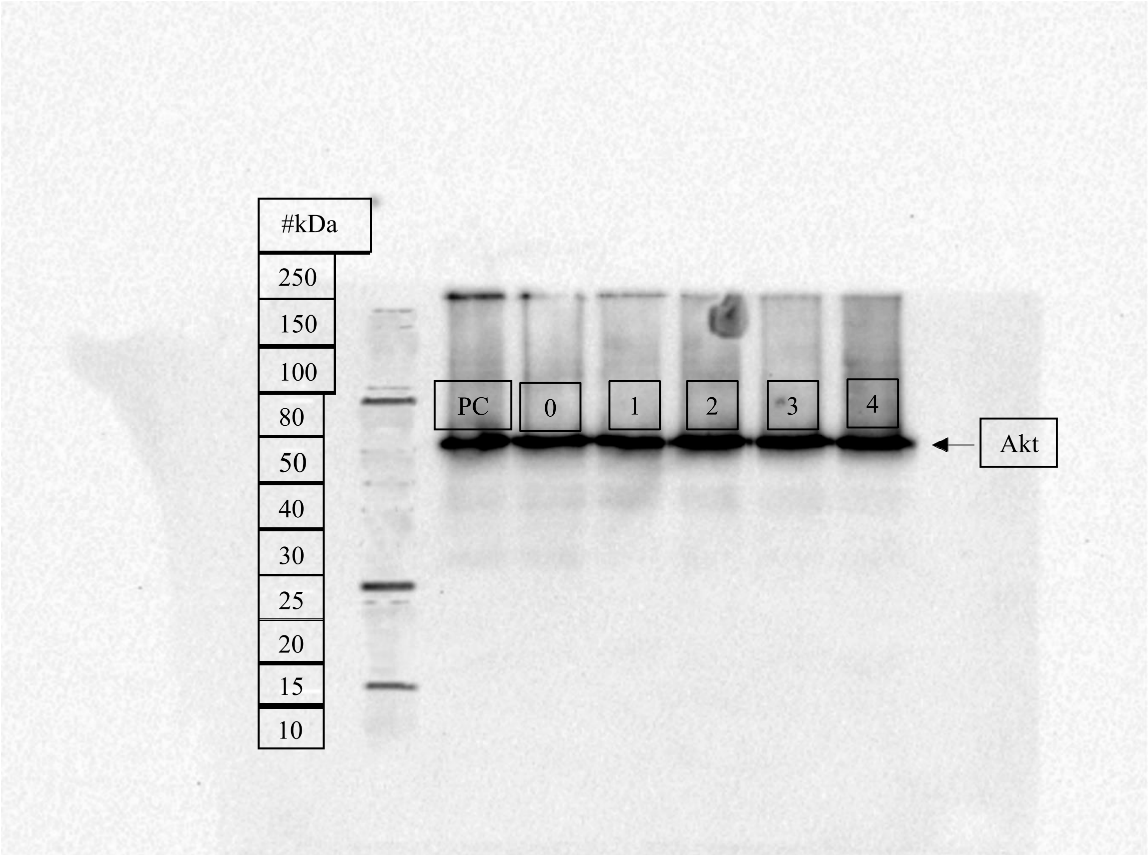

Figure S1. Western Blot membrane of Akt protein (56 kDa) detected with anti-Akt (1:1000, Cell Signaling C67E7). SDS page were transferred to nitrocellulose membranes (0.2 µm pore size; Bio-Rad) by semidry electroblotting (100 V, 2 hours). Membranes were incubated with an anti-rabbit secondary antibody (Cell Signaling 65-6120; 1:4000) and detection was performed using an enhanced chemiluminescence protocol (SuperSignal West Pico PLUS Chemiluminescent Substrate [ThermoFisher]). #Weight marker (kDa) used: Protein Ladder BioLabs p7703s, 10 to 250 kDa. Blot images, were converted to grayscale with ImageJ software (v. 1.52a, National Institute of Health, USA). PC: Positive control; 0: Control group; 1, 2, 3 and 4 – lugol treatments (30 minutes).

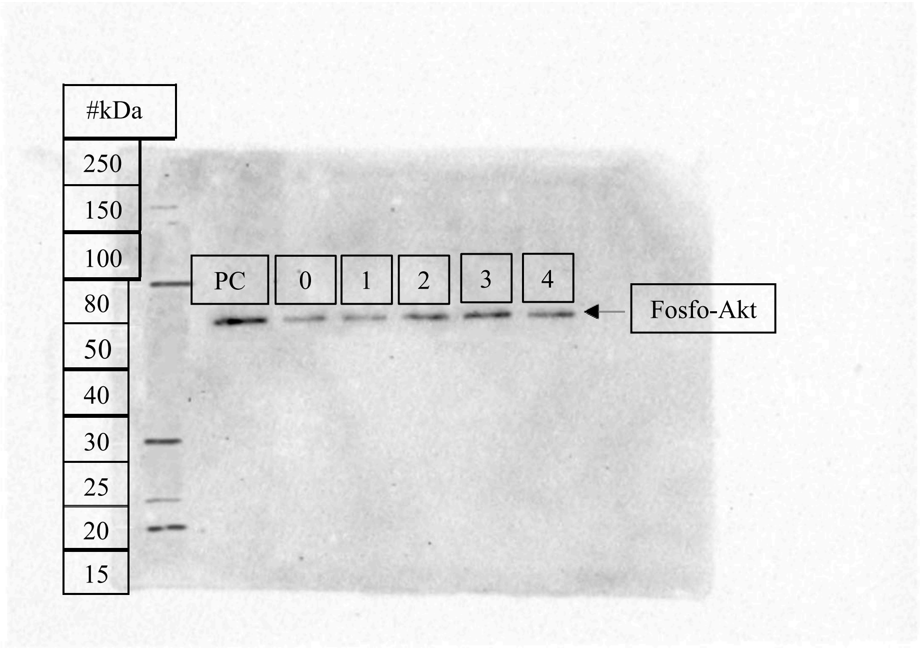

Figure S2. Western Blot membrane of Akt ser473 protein (56 kDa) detected with anti-fosfo-Akt (1:1000, Cell Signaling 9271). SDS page were transferred to nitrocellulose membranes (0.2 µm pore size; Bio-Rad) by semidry electroblotting (100 V, 2 hours). Membranes were incubated with an anti-rabbit secondary antibody (Cell Signaling 65-6120; 1:4000) and detection was performed using an enhanced chemiluminescence protocol (SuperSignal West Pico PLUS Chemiluminescent Substrate [ThermoFisher]). #Weight marker (kDa) used: Protein Ladder BioLabs p7703s, 10 to 250 kDa. Blot images, were converted to grayscale with ImageJ software (v. 1.52a, National Institute of Health, USA). PC: Positive control; 0: Control group; 1, 2, 3 and 4 – lugol treatments (30 minutes).

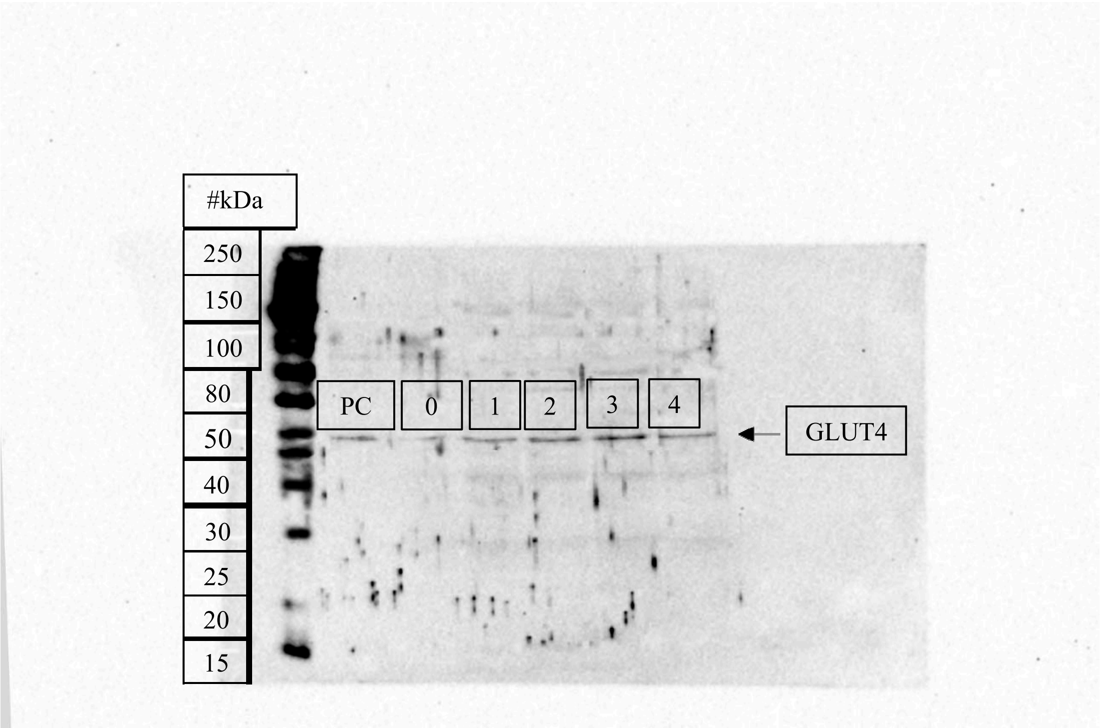

Figure S3. Western Blot membrane of GLUT4 protein (55 kDa) detected with anti-GLUT4 (1:2000, ab654 Abcam). SDS page were transferred to nitrocellulose membranes (0.2 µm pore size; Bio-Rad) by semidry electroblotting (100 V, 2 hours). Membranes were incubated with an anti-rabbit secondary antibody (Cell Signaling 65-6120; 1:4000) and detection was performed using an enhanced chemiluminescence protocol (SuperSignal West Pico PLUS Chemiluminescent Substrate [ThermoFisher]). #Weight marker (kDa) used: Protein Ladder BioLabs p7703s, 10 to 250 kDa. Blot images, were converted to grayscale with ImageJ software (v. 1.52a, National Institute of Health, USA). PC: Positive control; 0: Control group; 1, 2, 3 and 4 – lugol treatments (30 minutes).

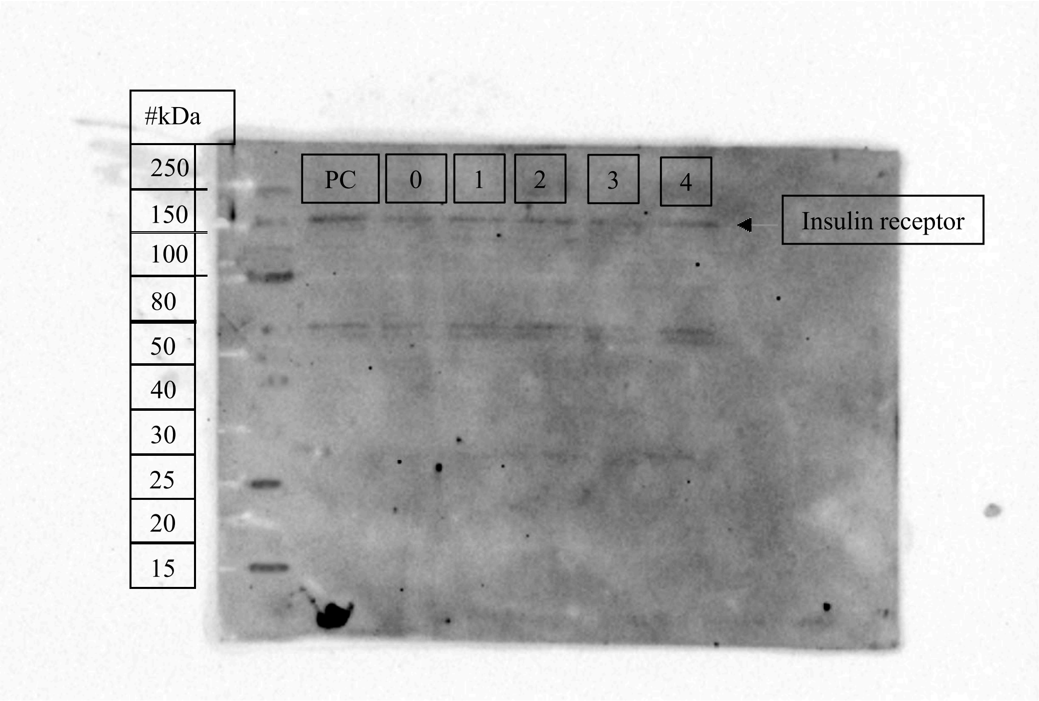

Figure S4. Western Blot membrane of insulin receptor protein (125 kDa) detected with anti-Insulin receptor alpha (1:2000, ab5500 Abcam). SDS page were transferred to nitrocellulose membranes (0.2 µm pore size; Bio-Rad) by semidry electroblotting (100 V, 2 hours). Membranes were incubated with an anti-rabbit secondary antibody (Cell Signaling 65-6120; 1:4000) and detection was performed using an enhanced chemiluminescence protocol (SuperSignal West Pico PLUS Chemiluminescent Substrate [ThermoFisher]). #Weight marker (kDa) used: Protein Ladder BioLabs p7703s, 10 to 250 kDa. Blot images, were converted to grayscale with ImageJ software (v. 1.52a, National Institute of Health, USA). PC: Positive control; 0: Control group; 1, 2, 3 and 4 – lugol treatments (30 minutes).

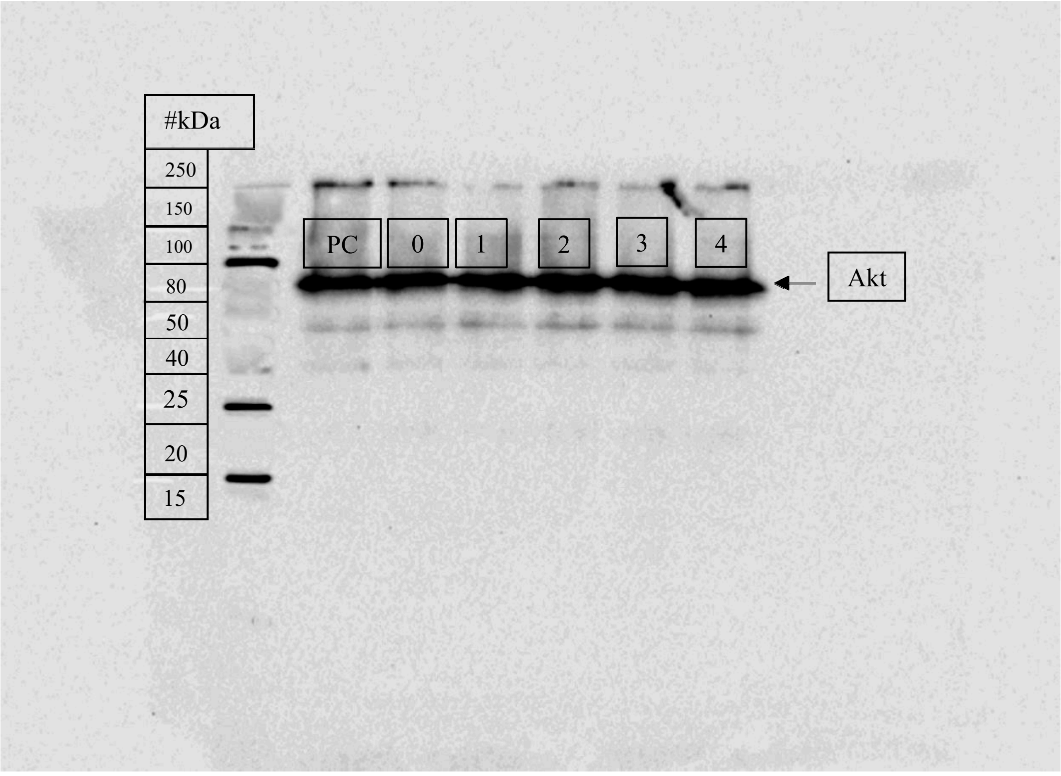

Figure S5. Western Blot membrane of Akt protein (56 kDa) detected with anti-Akt (1:1000, Cell Signaling C67E7). SDS page were transferred to nitrocellulose membranes (0.2 µm pore size; Bio-Rad) by semidry electroblotting (100 V, 2 hours). Membranes were incubated with an anti-rabbit secondary antibody (Cell Signaling 65-6120; 1:4000) and detection was performed using an enhanced chemiluminescence protocol (SuperSignal West Pico PLUS Chemiluminescent Substrate [ThermoFisher]). #Weight marker (kDa) used: Protein Ladder BioLabs p7703s, 10 to 250 kDa. Blot images, were converted to grayscale with ImageJ software (v. 1.52a, National Institute of Health, USA). PC: Positive control; 0: Control group; 1, 2, 3 and 4 – lugol treatments (6 hours).

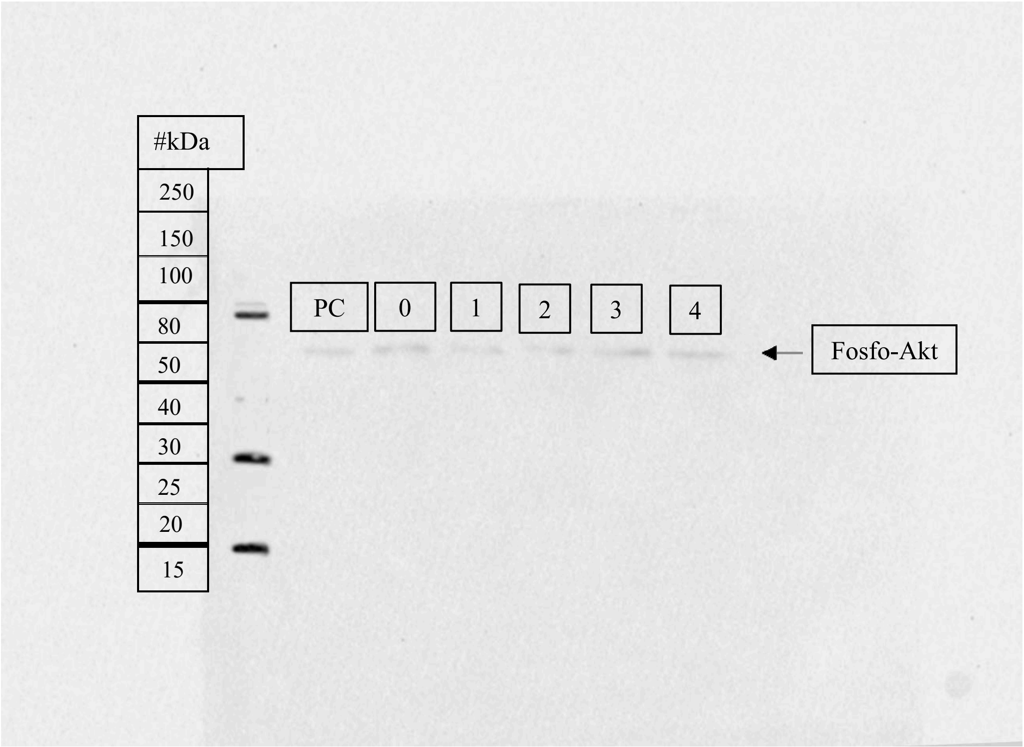

Figure S6. Western Blot membrane of Akt ser473 protein (56 kDa) detected with anti-fosfo-Akt (1:1000, Cell Signaling 9271). SDS page were transferred to nitrocellulose membranes (0.2 µm pore size; Bio-Rad) by semidry electroblotting (100 V, 2 hours). Membranes were incubated with an anti-rabbit secondary antibody (Cell Signaling 65-6120; 1:4000) and detection was performed using an enhanced chemiluminescence protocol (SuperSignal West Pico PLUS Chemiluminescent Substrate [ThermoFisher]). #Weight marker (kDa) used: Protein Ladder BioLabs p7703s, 10 to 250 kDa. Blot images, were converted to grayscale with ImageJ software (v. 1.52a, National Institute of Health, USA). PC: Positive control; 0: Control group; 1, 2, 3 and 4 – lugol treatments (6 hours).

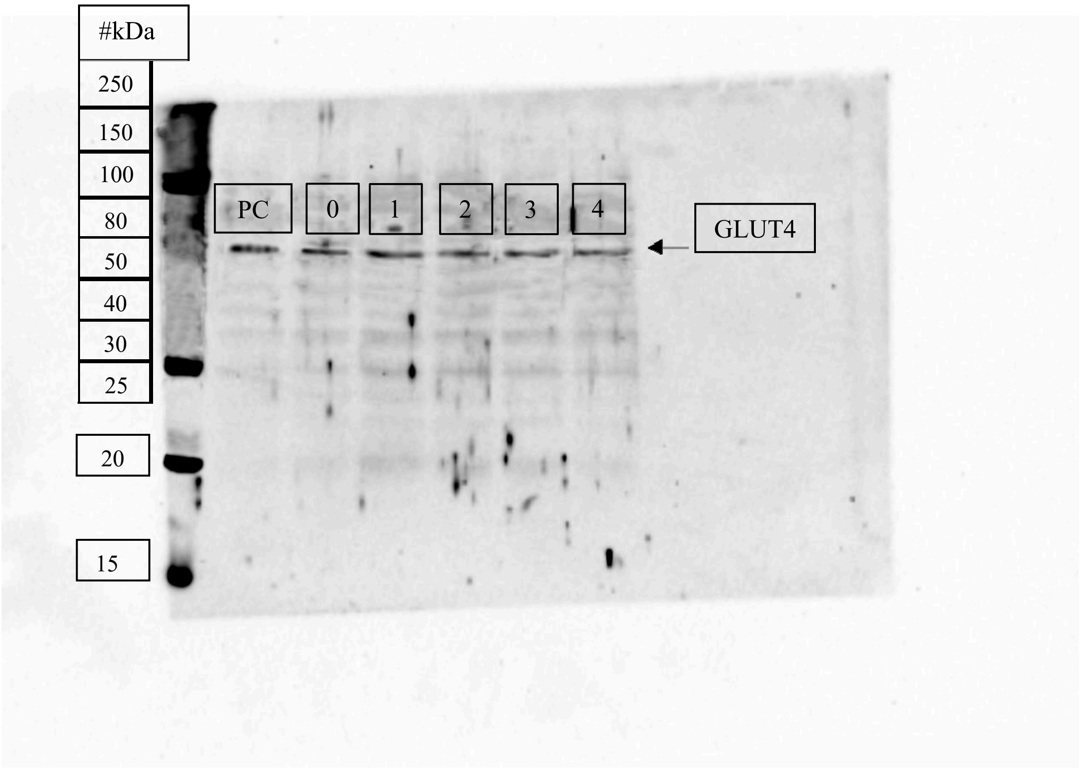

Figure S7. Western Blot membrane of GLUT4 protein (55 kDa) detected with anti-GLUT4 (1:2000, ab654 Abcam). SDS page were transferred to nitrocellulose membranes (0.2 µm pore size; Bio-Rad) by semidry electroblotting (100 V, 2 hours). Membranes were incubated with an anti-rabbit secondary antibody (Cell Signaling 65-6120; 1:4000) and detection was performed using an enhanced chemiluminescence protocol (SuperSignal West Pico PLUS Chemiluminescent Substrate [ThermoFisher]). #Weight marker (kDa) used: Protein Ladder BioLabs p7703s, 10 to 250 kDa. Blot images, were converted to grayscale with ImageJ software (v. 1.52a, National Institute of Health, USA). PC: Positive control; 0: Control group; 1, 2, 3 and 4 – lugol treatments (6 hours).

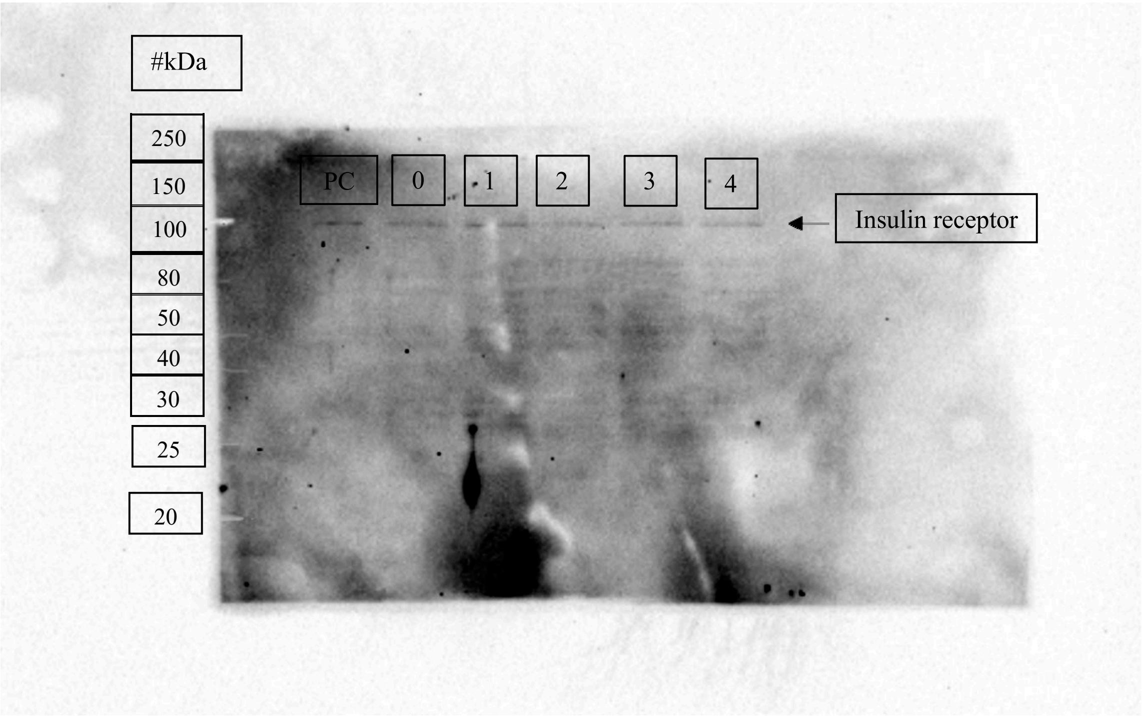

Figure S8. Western Blot membrane of insulin receptor protein (125 kDa) detected with anti-Insulin receptor alpha (1:2000, ab5500 Abcam). SDS page were transferred to nitrocellulose membranes (0.2 µm pore size; Bio-Rad) by semidry electroblotting (100 V, 2 hours). Membranes were incubated with an anti-rabbit secondary antibody (Cell Signaling 65-6120; 1:4000) and detection was performed using an enhanced chemiluminescence protocol (SuperSignal West Pico PLUS Chemiluminescent Substrate [ThermoFisher]). #Weight marker (kDa) used: Protein Ladder BioLabs p7703s, 10 to 250 kDa. Blot images, were converted to grayscale with ImageJ software (v. 1.52a, National Institute of Health, USA). PC: Positive control; 0: Control group; 1, 2, 3 and 4 – lugol treatments (6 hours).
